# *Pasteurellaceae* members with similar morphological patterns associated with respiratory manifestations in ducks

**DOI:** 10.14202/vetworld.2019.2061-2069

**Published:** 2019-12-26

**Authors:** Samah Eid, Sherif Marouf, Hefny Y. Hefny, Nayera M. Al-Atfeehy

**Affiliations:** 1 Department of Bacteriology, Reference Laboratory for Veterinary Quality Control on Poultry Production, Animal Health Research Institute, Agricultural Research Centre, Nadi El-Seid St., P.O. Box 246, Dokki, Giza 12618, Egypt; 2 Department of Microbiology, Faculty of Veterinary Medicine, Cairo University, Giza, Egypt; 3 Department of Poultry Diseases, Zagazig Provincial Laboratory, Animal Health Research Institute, Agricultural Research Centre, Sharkia, Egypt

**Keywords:** ducks, *Gallibacterium anatis*, *Mannheimia haemolytica*, *Pasteurella multocida*, *Riemerella anatipestifer*

## Abstract

**Aim:**

A total of 112 freshly dead ducks aged from 2 to 20 weeks old with a history of respiratory manifestations were investigated for the implication of *Pasteurellaceae* family members.

**Materials and Methods:**

Isolation and identification to the family level were conducted by conventional bacteriological methods, including microscopic examination and biochemical characterization. Identification to the species level was conducted by polymerase chain reaction (PCR) and analytical profile index (API) 20E kits.

**Results:**

Conventional bacteriological isolation and biochemical characterization revealed the infection of 16/112 examined birds with a prevalence rate of 14.3%. PCR confirmed the detection of *Pasteurellaceae* family conserved genes *Rpo*B and *Boot*z in 16/16 (100%) isolates. PCR was also used for genus and species identification of the isolated *Pasteurellaceae* members; the results revealed that 5/16 (31.3%) of isolates were *Gallibacterium anatis* and 2/16 of isolates (12.5%) were *Pasteurella multocida*. *Riemerella anatipestifer*, *Mannheimia haemolytica*, and *Avibacterium paragallinarum* were not detected by PCR. Biotyping by API 20E successfully identified 5/16 (31.3%) isolates that could not be typed by PCR and confirmed their belonging to *Pasteurella pneumotropica*. Neither the available PCR primer sets nor API 20E succeeded for species identification of 4/16 (25%) isolates. Antibiotic susceptibility profiling of isolates revealed that 16/16 (100%) of isolates demonstrated multidrug resistance (MDR) phenotypes. Moreover, 16/16 (100%) of isolates demonstrated a phenotypic resistance pattern to neomycin.

**Conclusion:**

Combined genotypic, phenotypic, biotyping, and virulence characterizations are required for laboratory identification of pathogenic *Pasteurellaceae*. Moreover, *P. multocida* was not the prevailed member implicated in respiratory problems in ducks as *P. pneumotropica*, *G. anatis*, and unidentified strains were involved with higher prevalence. Chloramphenicol and ampicillin demonstrated the highest *in vitro* effects on the studied *Pasteurellaceae*. Furthermore, the prevalence of multidrug-resistant isolates signified the demand to implement targeted surveillance in the ducks’ production sector, and MDR survey in poultry sectors in Egypt to apply effective control measures.

## Introduction

Rearing ducks are popular in Egypt at households and intensive commercial production systems as well. The popularity of ducks’ production in Egypt is encouraged by the fact that ducks are better adapted than chickens for environmental conditions, require less care, and are more resistant to diseases in addition to being of high and palatable meat and eggs [1]. Analysis of agricultural data demonstrated that ducks’ meat importation international record increased from 81 thousand tons to 187 thousand tons (+130%) in the past two decades as consumers mostly prefer meat and eggs of ducks and geese not only because of their taste but also for their high nutritional value and the fact that they contain optimum quantity of essential amino acids and fatty acids [2].

Although most *Pasteurellaceae* family members are considered commensals, some cause a variety of diseases in humans and animals, in this regard, pasteurellosis caused by *Pasteurella multocida* is proven to be contagious and endemic disease that causes high morbidity and mortality in domestic and wild birds. Furthermore, pasteurellosis is considered one of the major causes associated with high mortality in ducks [3].

Taxonomy of *Pasteurellaceae* is not adequately settled and is still under revision, in this instance taxonomy studies involved mainly phenotypic characterization and few scientific works have studied taxonomy to the genotypic level [4]. Since 1995, *Pasteurellaceae* family has been expanded from three to 13 genera through the use of new genetic-based classification and identification technologies, for example, genus *Gallibacterium* [5]. Recently, the three genera *Haemophilus*, *Actinobacillus*, and *Pasteurella* which originally formed the family, have been joined by new genera [6]. Currently, the family *Pasteurellace* ae comprises 57 named bacterial species that have been isolated from man and animals. The use of large and specific set of biochemical tests to identify and differentiate natural taxa of *Pasteurella* and related groups of organisms was described in several studies [7,8].

With the exception of *P. multocida*, limited studies are available with regard to the investigation of ducks’ infection with different genera and species of *Pasteurellaceae* family. Thus, fowl cholera caused by *P. multocida* remains one of the main problems of poultry worldwide and represents serious threats for ducks in Egypt [9,10].

For the past decade, antimicrobial resistance has become a problem of concern in human and veterinary medicines as being implicated in the failure of treatments’ protocols. Therefore, the proper determination of the resistance patterns represents a prerequisite for adapting successful control measures.

This study aimed to investigate the implication of *Pasteurellaceae* members as causative agents of respiratory diseases in ducks by applying comprehensive laboratory diagnostic tools using bacteriological, biochemical assays, and molecular approaches for differentiation between the phenotypic *Pasteurellaceae* variants to the genus and species levels. The study also aimed to investigate the antimicrobial resistance attributes of isolates.

## Materials and Methods

### Ethical approval

Albino mice used in the pathogenicity test were dealt with, according to OIE, Terrestrial Animal Health Code, 2018 [11].

### Sample collection

A total of 112 freshly dead ducks (Muscovy and Mallard) aged from 2 to 20 weeks old with a history of respiratory manifestations, high morbidity, and high mortality rates were transported under aseptic condition in iceboxes within maximum 24 h from commercial farms located at Hefna town, Belbes district, Sharkia Governorate, Egypt to Animal Health Research Provincial Laboratory in Zagazig city for postmortem examination and sampling from internal organs (liver, lung, heart, and spleen), freshly dead carcasses were kept in maintained refrigeration temperature at 5°C±2°C during the period elapsed from farm investigation till delivered to the laboratory. The study was applied during winter season 2018-2019.

### Postmortem examination

The carcasses that demonstrated different degrees of congestion in internal organs (liver, lungs, and spleen) with multiple, small necrotic foci and petechial hemorrhage on the coronary fat, thoracic air sacs, trachea, endocardium, and intestines were considered suspected for *Pasteurellaceae* infection; subsequently, internal organs (liver, lung, heart, and spleen) were collected under aseptic condition and transferred in iceboxes to reference laboratory for quality control on poultry production (RLQP), Dokki for laboratory diagnosis.

### Isolation, phenotypic, and biochemical identification

Samples were inoculated in brain heart infusion (BHI) broth, incubated at 37°C for 24 h. Loopful from broth culture was streaked onto blood agar plate (5% sheep blood), MacConkey agar plate and incubated at 37°C for 24 h.

Small circular convex glistening dewdrop like colonies, with or without hemolysis on 5% sheep blood agar plate, were considered suspected colonies for *Pasteurellaceae* family.

The isolated pure culture was smeared, stained with Gram and Leishman’s stains for microscopic examination and identification of characteristic bipolar isolates. Cultures were evaluated for oxidase activity, tryptophanase activity by indole test, catalase activity, urease activity, citrate utilization test, and H2S production, according to Florence *et al*. [12]. Isolates were also tested for fermentation of carbohydrates (mannitol, trehalose, sorbitol, glucose, mannose, fructose, xylose, arabinose, lactose, galactose, and dulcitol).

The isolates were identified on the basis of phenotypic, cultural and biochemical characteristics, combined with microscopic examination and morphological characteristics by Gram staining as Gram-negative coccobacilli, bipolarity by Leishman’s stain, according to Bisgaard *et al*. [13].

### Pathogenicity in mice

A loopful from each of 16 isolates was propagated in BHI broth for 18 h at 37°C, 0.1 ml from each broth culture was injected subcutaneously into two albino mice (Female BALB/c mice at 6-10 weeks of age), and two mice were inoculated with 0.1 ml of sterile BHI broth as negative control, according to Ramdani *et al*. [14]. The challenged 32 and the two control mice were observed for 48 h; mortality was recorded. Heart blood smears and impression smears of spleen, liver, and lung were applied from dead mice, stained with Leishman’s stain, and streaked on 5% sheep blood agar for reisolation.

### Antimicrobial susceptibility profiles

Antimicrobial susceptibility profiles of isolates were studied by disk diffusion method against 11 antimicrobial agents of the most commonly used in the domestic field for the treatment of respiratory manifestations in ducks and that belong to 8 antimicrobial groups as follows: Spectinomycin (SH-100 µg), nalidixic acid (NA-30 µg), penicillin (P-10 µg), cefotaxime (CTX-30 µg), trimethoprim (TR-5 µg), ampicillin (AMP-10 µg), oxytetracycline (OT-30 µg), chloramphenicol (C-30 µg), nitrofurantoin (F-300 µg), neomycin (N-30 µg), and levofloxacin (LEV-5 µg), and the test was performed and interpreted, according to CLSI-M45 A2, [15].

### Confirmation and species identification of isolates by polymerase chain reaction (PCR)

#### DNA extraction

Genomic DNA extract was obtained using (Gene JET Genomic DNA purification Kit Thermo scientific). DNA concentration was determined by a spectrophotometer at 260/230 nm. The used primers, PCR conditions, and thermal profiles are listed in Table-1 [4,16-22].

**Table-1:** Primers’ sequences and cycling conditions for avian *pasteurellaceae.*

Test target	Gene	Amplicon size (bp)	Primer sequence (5′-3′)	Thermal profile	References
*Pasteurellaceae* family	*PasRpob-*L	560	(F) 5′GCAGTGAAAGARTTCTTTGGTTC3′ (R) 5′GTTGCATGTTNGNACCCAT3′	3 min denaturation at 94°C followed by 35 cycles: 94°C for 30 s, 54°C for 30 s, 72°C for 30 s, final extension step at: 72°C for 7 min	[4]
	*Bootz*	533	(F) 5′CAT AAG ATG AGC CCA AG 3′ (R) 5′GTC AGT ACA TTC CCA AGG 3′	4 min denaturation at 95°C followed by 40 cycles: 94°C for 60 s, 55°C for 60 s, 72°C for 60 s, final extension step at: 72°C for 4 min	[16]
*Riemerella anatipestifer*	16S rRNA	665	(F) 5′CAGCTTAACTGTAGAACTGC 3′ (R) 5′TCGAGATTTGCATCACTTCG 3′	5 min denaturation at 95°C followed by; 25 cycles at 94°C for 30 s; 54°C for 50 s; 72°C for 1 min; final 7 min at 72°C	[17]
	669 A	669	(F) 5′TTACCGACTGATTGCCTTCTAG-3′ (R) 5′AGAGGAAGACCGAGGACATC-3′	4 min denaturation at 95°C followed by; 35 cycles: 94°C for 1 min, 55°C for 1 min, 72°C for 1 min, with a final extension step at 72°C for 7 min	[18]
*Gallibacterium anatis*	16S RNA and 23S RNA	1032	(F) 5′TATTCTTTGTTACCACGG3′ (R) 5′GGTTTCCCCATTCGG3′	5 min denaturation at 95°C followed by; 35 cycles at 94°C for 30s; 55°C for 40s; 72°C for 1-2 min; final 10 min at 72°C	[19]
*Mannheimia haemolytica*	*Rpt*2	1022	(F) 5′GTTTGTAAGATATCCCATTT3′ (F) 5′CGTTTTCCACTTGCGTGA3′	3 min denaturation at 95°C followed by; 30 cycles at 95°C for 30 s; 95°C for 1 min; 48°C for 1 min; extension at 72°C for 30 s	[20]
*Pasteurella multocida*	*KMT*	460	(F) 5′GCTGTAAACGAACTCGCCC 3′ (R) 5′ATCCGCTATTTACCCAGTGG 3′	4 min denaturation at 95°C followed by 30 cycles of: 95°C for 1 min, 55°C for 1 min, 72°C for 1 min final extension at 72°C for 9 min	[21]
*Avibacterium paragallinarum*	*HPG-2*	500	(F) 5′TGAGGGTAGTCTTGCACGCGAAT3′ (R) 5′CAAGGTATCGATCGTCTCTCTACT3′	4 min denaturation at 95°C followed by 30 cycles of: 94°C for 1 min, 63°C for 1 min, 72°C for 30s. Final extension at 72°C for 10 min	[22]

#### PCR amplification

PCR reaction was performed in a gradient thermal cycler (1000 S Thermal cycler Bio-RAD USA). The reaction mixture with a total volume of 50 µl; was consisted of 25 µl dream green PCR Mix (Dream Taq Green PCR Master Mix (2×) Thermo Scientific Company- cat. No.K1080, USA.), 3 µl target DNA, 1 µl of each primers with a concentration of 10 p mole/µl, the mixture was completed to 50 µl with PCR water. Positive control strain: Standard strain confirmed and provided by ISO 17025 Biotechnology Department AHRI, Dokki Giza, Egypt, was used, negative control: *Escherichia coli* reference strain: NCTC 12241/ATCC 2522 was used as a negative control.

### Analysis of PCR products

PCR products were separated by electrophoresis on 1% agarose gel (Applichem, Germany, GmbH) in 1× TBE buffer at room temperature using gradients of 5V/cm. For gel analysis, 15 µl of products were loaded in each gel slot. Gel pilot 100 bp plus ladder (Qiagen, Germany, GmbH) was used to determine the fragment size. The gel was photographed by a gel documentation system (Alpha Innotech, Biometra).

## Results

### Prevalence rate of Pasteurellaceae family

Results of isolation and biochemical characterization revealed that *Pasteurellaceae* family members were isolated from 16 out of 112 examined birds with a prevalence rate of 14.3%, a higher prevalence rate of 8/28 (28.6%) was detected in the age group of <6 weeks old, Table-2. The results of isolation also demonstrated that the prevalence rate was higher in Muscovy breed (6 out of 39 examined ducks) compared with Mallard breed (10 out of examined 73) with prevalence rates of 15.4% and 13.7%, respectively.

**Table-2:** Prevalence rate of *Pasteurellaceae* family members with regard to birds’ age group.

Age group	Number of birds		Prevalence rate (%)

	Examined birds	Infected birds	
<6 weeks old	28	8	28.6*
6-12 weeks old	48	5	10.4*
>12 weeks old	36	3	8.3*
Total	112	16	14.3**

* The prevalence rate was calculated based on the number of examined birds in each age group,

** The prevalence rate was calculated based on the total number of examined birds

### Pathogenicity test in albino mice

The study of pathogenicity test in challenged albino mice (Female BALB/c mice at 6-10 weeks of age) for the 16 *Pasteurellaceae* isolates resulted in 100% mortality of mice within 48 h, whereas, no mortality was recorded in the control mice. Smears of heart blood, spleen, liver, and lung from dead mice revealed the identification of characteristic bipolar microorganisms with Leishman’s stain.

Antimicrobial susceptibility profiling of isolates was tested against 11 of the most field used antimicrobial agents that belong to eight antimicrobial groups, the results revealed that 16/16 (100%) of isolates demonstrated phenotypic resistance patterns against neomycin, while none of the isolates demonstrated resistance to nitrofurantoin. Moreover, 14/16 (87.5%) of isolates demonstrated phenotypic resistance to spectinomycin, penicillin G, and 13/16 (81.3%) of isolates demonstrated phenotypic resistance to oxytetracycline, and 12/16 (75%) of isolates demonstrated resistance to nalidixic acid, 9/16 (56.3%) to levofloxacin. Moreover, resistance rates to cefotaxime, trimethoprim, ampicillin, and chloramphenicol were 7/16 (43.8%), 6/16 (37.5%), 4/16 (25%), and 2/16 (12.5%), respectively. The results also revealed that 16/16 (100%) of isolates demonstrated multidrug resistance (MDR) phenotypes to antimicrobial agents that belong to three and more antimicrobial categories as interpreted by CLSI-M45 A2, [15]. The results also revealed that 8/16 (50%), 3/16 (18.8%), and 2/16 (33.3%) of isolates demonstrated MDR to antimicrobial agents that belong to 5, 4, and 6 antimicrobial groups, respectively, Table-3.

**Table-3:** Antimicrobial resistance patterns of *Pasteurellaceae* isolates.

Antimicrobial category	Quinolones		Penicillin		Cephalosporin	Diaminopyrimidine	Tetracycline	Macrolide	Sulfonamides	Aminoglycosides		Multidrug resistance
									
Isolate NO	Nalidixic A 30 µg	Levofloxacin 5 µg	Penicillin G 10 µg	Ampicillin 10 µg	Cefotaxime 30 µg	Trimethoprim 5 µg	Oxytetracycline 30 µg	Chloramphenicol 30 µg	Nitrofurantoin 300 µg	Neomycin 30 µg	Spectinomycin 100 µg	
1	S	S	R	R	R	R	R	S	S	R	S	5
2	S	R	R	S	S	S	S	S	S	R	R	3
3	R	R	R	S	S	S	R	S	S	R	R	4
4	R	R	R	S	R	S	R	S	S	R	R	5
5	S	R	R	S	S	R	R	S	S	R	R	5
6	R	S	R	S	R	S	R	S	S	R	R	5
7	R	S	R	S	S	R	R	S	S	R	R	5
8	R	R	R	S	S	S	S	S	S	R	S	3
9	R	R	R	R	R	S	R	R	S	R	R	6
10	R	S	R	R	R	R	R	S	S	R	R	6
11	R	S	R	S	R	S	R	S	S	R	R	5
12	R	R	S	S	S	R	R	S	S	R	R	4
13	R	R	R	S	S	R	R	S	S	R	R	5
14	R	S	R	R	R	S	R	S	S	R	R	5
15	R	S	S	S	S	S	R	R	S	R	R	4
16	S	R	R	S	S	S	S	S	S	R	R	3
Resistant isolates	12/16	9/16	14/16	4/16	7/16	6/16	13/16	2/16	0	16/16	14/16	
Resistance rate (%)	75	56.3	87.5	25	43.8	37.5	81.3	12.5	0	100	87.5	

### PCR for confirmation and species identification

**Figure-1:**
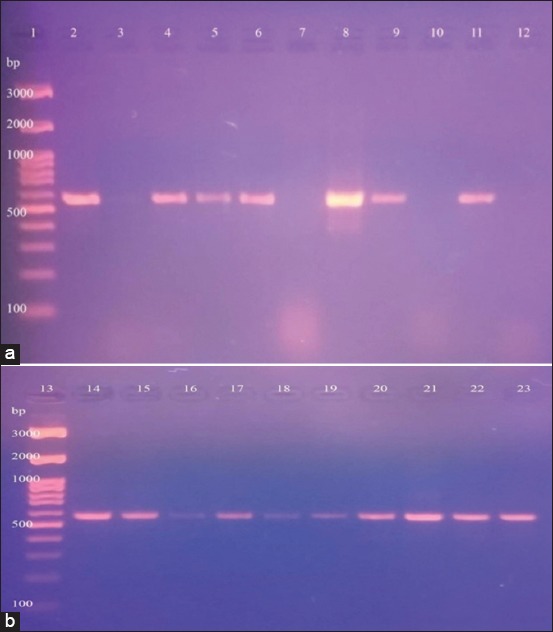
(a and b) Lanes 1,13: Gel pilot 100 bp plus ladder, Lane 23: Positive control, Lanes 2, 4, 5, 6, 8, 9, 11, 14-22: Positive specific amplicon for *PRpob*-L gene at 560 bp, Lanes 3, 7, 10: Negative with no amplicon.

**Figure-2:**
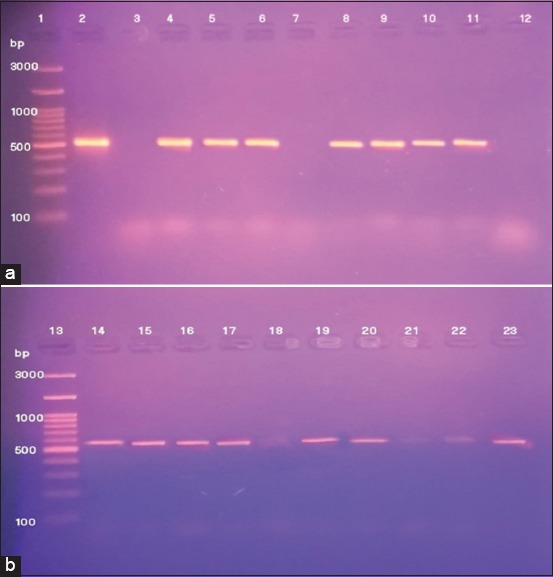
(a and b) Lanes 1, 13: Gel pilot 100 bp plus ladder, Lane 23: Positive control, Lanes 2, 4, 5, 6, 8, 9, 11, 14-22: Positive specific amplicon for *Boot*z gene at 533 bp, Lanes: 3, 7, 10 negative with no amplicon.

PCR confirmed that the 16 isolates belonged to family *Pasteurellaceae* by the detection of the specific conserved genes, *Pas Rpob-*L gene, and *Bootz* gene, Figures-1a and b, 2a and b, respectively. PCR testing for the presence of *KMT* gene also confirmed that 2/16 (12.5%) of isolates belonged to *P. multocida*. PCR testing for the detection of 16S RNA and 23S RNA genes of *Gallibacterium anatis* confirmed the positivity of 5/16 (31.3%) of isolates. However, PCR failed to detect *Rpt*2 gene specific for *Mannheimia haemolytica*, PCR also failed to detect 669 A gene and 16S rRNA gene specific for *Riemerella anatipestifer*, furthermore, PCR failed to detect *HPG-*2 gene specific for *Avibacterium paragallinarum*, respectively.

### Biotyping of isolates by analytical profile index (API) 20E kit

API 20E test was applied for biotyping and identification of the nine isolates that were not identified by the available used PCR primer sets, the result revealed that 5/16 (31.3%) of isolates were identified as *Pasteurella pneumotropica*. Moreover, API 20 E failed to identify 4/16 (25%) of *Pasteurellaceae* family members involved in the study.

## Discussion

*Pasteurellaceae* family involves many pathogenic and non-pathogenic species. Meanwhile, several species are considered opportunistic secondary invaders that cause infections under predisposing circumstances; other species can play the role of primary pathogens that are capable of causing severe diseases in many animals [23]. In this regard, fowl cholera caused by *P. multocida* is considered the most important infectious diseases in birds, especially waterfowls [24].

In the present study, conventional bacteriological and biochemical methods were used to examine 112 freshly dead ducks with a history of respiratory manifestations and postmortem gross lesions for *Pasteurellaceae* infection, the results revealed the isolation of *Pasteurellaceae* from 16 out of 112 examined ducks with a prevalence rate of 14.3%. Many studies accounted for conventional bacteriological methods and microscopic examination for demonstration of Gram-negative coccobacilli with a bipolar morphological appearance using Gram stain and Leishman’s stain for the identification of *P. multocida* [25,26].

The results revealed that the highest prevalence of *Pasteurella* infection was observed in young ducklings of <6 weeks old as eight out of 28 (28.6%) examined birds were positive for *Pasteurella* isolation, the results also revealed that two out of 16 *Pasteurella* isolates were identified by PCR as *P. multocida* and were isolated from the same age group. Moreover, 13 out of 16 (81.25%) ducklings of <12 weeks old were positive to *Pasteurella* infection. These results were in agreement with the published scientific conclusions that birds younger than 11 weeks old were more prone to *Pasteurella* infection and that susceptibility of ducks most likely peaks somewhere between 4 and 11 weeks of the bird’s age [27,28].

Studying the association of breeds’ susceptibility indicated that Muscovy breed encountered more infection compared to Mallard breed with prevalence rates of 6/39 (15.4%), 10/73 (13.7%), respectively. Similar studies recorded that Muscovy ducklings were found more susceptible to *P*. multocida infection compared to Pekin ducklings [29]. In the same regard, researches attributed the difference in immune response observed among different breeds of ducks to the breeds’ response toward environmental stressors as excitement, injury, climate changes, and nutrition [24].

*P. multocida* is considered as the causative agent of fowl cholera, the infectious disease of major economic significance for several animal species worldwide. This bacterial agent is also known to infect over 100 species of wild birds and causes recurrent epizootics that can kill tens of thousands of birds in a single event, with little warning [30]. Meanwhile, most of the published surveillance that approached respiratory diseases in ducks focused primarily on detecting *P. multocida* and *M. haemolytica*, the studies were not informative for investigating and managing other family members implicated as causative agents in respiratory diseases.

The current study targeted the identification of *Pasteurellaceae* members isolated from freshly dead ducks with a history of respiratory manifestation to the genus and species level using the available laboratory diagnostic tools.

The results revealed that the prevalence rates of *Pasteurellaceae* family members and *P. multocida* were (14.3%) and (1.7%), respectively. Higher prevalence rates for *P. multocida* (10.4% and 25.2%) from apparently healthy and diseased ducks were reported by other scientific studies, which concluded that *P. multocida* is the most predominant microorganism isolated from apparently healthy and diseased ducks followed by *E*. *coli* and staphylococci [9].

The results of the current study revealed that in addition to *P. multocida* other members of *Pasteurellaceae* family were implicated as causative agents in the investigated cases of respiratory manifestations and that *P. multocida* was not the prevailed isolated species. This result was confirmed by biochemical characterization and by PCR. Moreover, the pathogenicity of 16/16 *Pasteurellaceae* isolates was confirmed by applying bioassay in mice as an approved approach to test their pathogenicity [31]. Bioassay resulted in death of 32/32 (100%) of I/P injected mice. Pathogenicity of non *P. multocida* isolates was attributed to the ability of normal commensal bacteria to encounter pathogenicity concordant with some changes in the host status [32].

PCR is a test that can be used for the identification of organisms at any level; strain, species, genus or members of a domain just by using specific primer sequence. Moreover, PCR was considered the most promising approach for detecting infection with *Pasteurellaceae* [33,34]. In the present study, PCR was used to confirm the results of conventional bacteriological isolation and biochemical characterization. PCR targeted the detection of *Pasteurellaceae* family specific conserved genes *Rpo*B gene (Beta-subunit of DNA-dependent RNA-polymerase) and *Bootz* R gene, the results confirmed that 16 out of 16 (100%) isolates belonged to *Pasteurellaceae* family and produced the specific PCR products at 560-bp and at 533-bp, respectively. These results were in accordance with the scientific recommendation for the use of *Rpo*B gene for precise diagnosis, particularly in cases where phenotypic identification is difficult [35]. Moreover, the presence of phenotypic *Pasteurellaceae* variants makes it impossible to be correctly identified even by applying extended phenotypic characterization. Accordingly, the application of 16S rDNA gene sequencing was proposed to allow the correct identification of such variants [5].

Meanwhile, PCR identified two out of 16 isolates (12.5%) as *P. multocida*, attempts to identify the rest of 14 isolated *Pasteurellaceae* members to the genus and species level using PCR did not reveal the detection of *A. paragallinarum* (formerly *Haemophilus paragallinarum*) [36], *R. anatipestifer*, or *M. haemolytica* (formerly *Pasteurella haemolytica*). On the other hand, PCR successfully identified five out of 16 (31.3%) isolates as *G. anatis*. In this regard, growing demands were raised to apply more studies about *G. anatis* and its poorly understood growth kinetics, exact host-pathogen interplay, pathogenesis, and the switch from a normal inhabitant of healthy bird to a pathogen causing disease and mortality, virulence factors under more biologically relevant circumstances, and vaccines’ production [37,38].

Studies referred to the failure to isolate *M*. *haemolytica* to the higher concentration of iron required for its growth, the fastidious nature, the mesophilic nature, and the atmospheric requirements for facultative anaerobes and microaerophiles [6].

*R. anatipestifer* infection in ducklings produces a contagious disease, known as new duck disease or riemerellosis, during earlier periods it was misidentified as *P. multocida* due to their morphological and cultural similarities [39]. Trials were made by many researchers to isolate and identify *R. anatipestifer* as an important disease agent causing severe economic losses in ducks’ industry. Similar to the current result some researches failed to detect *R. anatipestifer* [40]. In the same instance, other studies confronted difficulties with *R. anatipestifer* identification [41]. Furthermore, and with regard to the failure to detect *R. anatipestifer* in the current study conducted in Winter 2018, other studies reported low incidence of *R. anatipestifer* in Winter in contrast to the high incidence of *R. anatipestifer* observed mainly during summer season with limited maximum morbidity and mortality rates in the period elapses from June to July and at the age group of 8-10 weeks old [42].

API 20E kit set was applied as a trial to identify the nine out of 16 isolates that could not be identified by the available PCR sets of primers. In this instance, API 20E system successfully identified 5/16 (31.3%) as *P. pneumotropica* while failed to identify four isolates among the isolated *Pasteurellaceae*.

The identification of *Pasteurellaceae* to the species level by API 20E system solely is somehow unreliable due to the existence of taxa that are not included in the API database and due to the limited diagnostic agreement between laboratories studying the same strains [43], this conclusion supported the current finding as the applied API 20E set demonstrated limitations in the identification of all the tested isolates and successfully identified only 5/9 tested isolates (*P. pneumotropica*) while failed to biotype 4/9 isolates. Although some studies considered *P. multocida* and *P. pneumotropica* of greater pathogenic importance than other *Pasteurellaceae*, other studies considered this conclusion unjustified [44].

Many reports signified the importance of precise differentiation between *Pasteurellaceae* members to avoid excess considerable expenses caused by false or delayed diagnosis and to allow precise and rapid identification especially in case of infection by *R. anatipestifer* thus, mistaking it for *P. multocida* lead to exaggerated untrue consternation of fowl cholera, while confounding it with nonpathogenic *Pasteurellaceae* results in delayed diagnosis of *Anatipestifer* syndrome [45].

Reviewing the scientific study concerned with the isolation and identification of *Pasteurellaceae* members referred these common diagnostic problems mainly to poor growth nature of this family members on artificial media and to subsequent overgrowth by other bacterial species even before identification can be initiated, the scientific studies also referred to the difficulties represented by phenotypic differentiation and identification, and the lack of reliable commercial identification system.

In the current study, phenotypic antimicrobial resistance profiles of *Pasteurellaceae* isolates were investigated against 11 antimicrobial agents that belong to eight antimicrobial categories, and the results revealed that 16/16 (100%) of studied isolates demonstrated phenotypic MDR attributes against three and more antimicrobial groups. The results also demonstrated that 100% of isolates demonstrated resistance against neomycin, while all 16/16 (100%) isolates were susceptible to nitrofurantoin. Moreover, 14/16 (87.5%) of isolates demonstrated phenotypic resistance to spectinomycin and penicillin G, 13/16 (81.3%) of isolates demonstrated phenotypic resistance to oxytetracycline, 12/16 (75%) of isolates demonstrated resistance to nalidixic acid, and 9/16 (56.3%) to levofloxacin. Moreover, resistance rates to cefotaxime, tetracycline, ampicillin, and chloramphenicol were 7/16 (43.8%), 6/16 (37.5%), 4/16 (25%), and 2/16 (12.5%), respectively. The MDR phenotypes observed among isolates may be attributed to the misuse of antimicrobial agents at the domestic field level. This finding accorded with reports published on the emergence of antimicrobial resistance among several organisms belonging to *Pasteurellaceae* family including *G. anatis* isolates [46].

Although nitrofuran is effective antimicrobial agent, its use in food animals was banned in the EU since 1995 as considered of carcinogenic effect [47,48]. Investigation on the resistance profiles of the circulating *Pasteurellaceae* members to nitrofurantoin was conducted by the current study and fortunately the results revealed the sensitivity of 16/16 (100%) of the tested isolates, thus can be considered indicative to compliance of field stakeholders (farm owners and veterinarians) to restrictions applied on the field use of this banned dangerous antimicrobial agent.

In contrast to the resistance phenotypes observed among *Pasteurellaceae* isolates in the current study, susceptibility of avian *P. multocida* to chloramphenicol, gentamycin, tetracycline, penicillin G, streptomycin, sulfonamide, and trimethoprim were recorded by other studies [49]. In the same regard, an Egyptian study recorded that enrofloxacin, norfloxacin, and ciprofloxacin were the most effective antibiotics against *P. multocida* isolated from ducks [9]. Moreover, lower resistance rates of *P. multocida* to ampicillin, cephalothin (<5%), and tetracycline (6.2%) were recorded [50].

In accordance with the current results, resistance rates of *P. multocida* isolated from ducks to penicillin G (100%), ampicillin (55%), and tetracycline (10%) were recorded [51]. Furthermore, some studies recorded phenotypic resistance of avian *P. multocida* isolates to enrofloxacin (23.21%) and to sulfaquinoxaline (76.79%), in addition to the observed multi-resistance in (19.64%) of isolates [52].

Recent study also reported high resistance rates demonstrated by *G. anatis* isolated from chickens and turkeys to sulfadimethoxine (93.3%), spectinomycin (93.3%), and oxytetracycline (80.0%), respectively [53]. Concerns were raised on the widespread MDR and substantial antigenic variation among strains of *G. anatis* as a major challenge that hinder treatment with antimicrobials [54,55].

## Conclusion

The study declared that laboratory diagnosis by conventional bacteriological and biochemical methods for the identification and differentiation between *Pasteurellaceae* family members was not sufficient for reliable genus and species identification, and discrimination. Thus, it signified the importance of applying combined bacteriological, biochemical, genetic, and virulence studies for the differentiation between *Pasteurellaceae* family members to provide clear epidemiological data that contribute in the effective control of pasteurellosis. Moreover, it is recommended to apply targeted survey on *Pasteurellaceae* in ducks’ production sector and investigate the resistance and virulence circulating profiles for the provision of valuable insights into respiratory problems and apply effective control measures in ducks’ farms in Egypt.

## Authors’ Contributions

SE designed the study, applied bacteriological examinations, Wrote and revised the manuscript and manage correspondence. SM applied PCR testing. HYH collected the samples and applied postmortem examinations. NMA applied bacteriological examinations and wrote the manuscript. All authors read and approved the final manuscript.
